# Identification and Validation of a Novel Genomic Instability-Associated Long Non-Coding RNA Prognostic Signature in Head and Neck Squamous Cell Carcinoma

**DOI:** 10.3389/fcell.2021.787766

**Published:** 2022-01-20

**Authors:** Yun Chen, Yaqiong Zhao, Ruohuang Lu, Han Zhao, Yue Guo

**Affiliations:** ^1^ Department of Stomatology, The Second Xiangya Hospital, Central South University, Changsha, China; ^2^ Department of Stomatology, The Third Xiangya Hospital, Central South University, Changsha, China; ^3^ Department of Ophthalmology, Eye, Ear, Nose and Throat Hospital of Fudan University, Shanghai, China; ^4^ Laboratory of Myopia, NHC Key Laboratory of Myopia (Fudan University), Chinese Academy of Medical Sciences, Shanghai, China; ^5^ Shanghai Key Laboratory of Visual Impairment and Restoration, Fudan University, Shanghai, China

**Keywords:** genomic instability, head and neck squamous cell carcinoma, long non-coding RNA, prognostic signature, survival

## Abstract

**Background:** Head and neck squamous cell carcinoma (HNSCC) is one of the most aggressive malignant cancers worldwide, and accurate prognostic models are urgently needed. Emerging evidence revealed that long non-coding RNAs (lncRNAs) are related to genomic instability. We sought to identify and validate a genomic instability-associated lncRNA prognostic signature to assess HNSCC patient survival outcomes.

**Methods:** RNA-sequencing data, somatic mutation files, and patient clinical data were downloaded from The Cancer Genome Atlas database. A total of 491 patients with completely clinical files were randomly divided into training and testing sets. In the training set, genomic instability-associated lncRNAs were screened through univariate Cox regression analyses and least absolute shrinkage and selection operator regression analyses to build a genomic instability-associated lncRNA signature (GILncSig). In addition, time-dependent receiver operating characteristic (ROC) curve, Kaplan-Meier survival curve, and clinical stratification analyses were used to evaluate the signature’s reliability. Finally, *in situ* hybridization experiments were performed to validate GILncSig expression levels between adjacent non-tumor tissues and tumor tissues from HNSCC patients.

**Results:** Four genomic instability-associated lncRNAs (AC023310.4, AC091729.1, LINC01564, and MIR3142HG) were selected for the prognostic signature. The model was successfully validated using the testing cohort. ROC analysis demonstrated its strong predictive ability for HNSCC prognosis. Univariate and multivariate Cox analyses revealed that the GILncSig was an independent predictor of prognosis. HNSCC patients with a low-risk score showed a substantially better prognosis than the high-risk groups. The *in situ* hybridization experiments using human HNSCC tissue revealed high GILncSig expression in HNSCC tissues compared with adjacent non-tumor tissues.

**Conclusion:** We developed a novel GILncSig for prognosis prediction in HNSCC patients, and the components of that signature might be therapeutic targets for HNSCC.

## Introduction

Head and neck cancer is the sixth most common tumor around the world and is also the most lethal with more than 450,000 annual deaths (Johnson et al., 2020). Head and neck squamous cell carcinoma (HNSCC) is the most common pathological type (Shield et al., 2017), and the current standard of treatment is surgery followed by chemotherapy plus radiation (Johnson et al., 2020). However, the 5-years survival rate for HNSCC has not improved significantly over the past 30 years. Cervical lymph node metastasis, local recurrence, and resistance to conventional chemotherapy and radiotherapy often occur in HNSCC patients with advanced-stage disease (Pisani et al., 2020). The current traditional prognostic methods for HNSCC patients are based on clinicopathological parameters including tumor size, nodal status, and the existence of metastases. However, many patients with the same tumor stage have different survival outcomes (Marur and Forastiere, 2016). Despite advances in diagnostic techniques, most patients are diagnosed with an advanced stage HNSCC with a low curative ratio and have a prognosis (Ferlay et al., 2015; Vendrely et al., 2018). There is an urgent need to identify valuable biomarkers to predict the prognosis of HNSCC patients.

Genomic instability is considered one of the hallmarks of human cancers (Hanahan and Weinberg, 2000; Hanahan and Weinberg, 2011) and is defined as the increased probability of acquiring chromosomal aberrations due to defects in processes such as DNA repair, DNA damage, replication, or chromosome segregation (Lord and Ashworth, 2012; Tubbs and Nussenzweig, 2017). Based on the level of genomic disruption, genomic instability is typically subdivided into three categories: nucleotide, microsatellite, and chromosome (Pikor et al., 2013). Cancer genomic instability contributes to genetic heterogeneity within tumors and the wide range of phenotypic diversity observed in patients. Extensive chromosome rearrangements in cancer genomes can facilitate oncogenic progression (Hanahan and Weinberg, 2011), but the underlying mechanisms of genomic instability have not been fully explored. Recent evidence suggests that genomic instability is related to tumor initiation, progression, and survival (Suzuki et al., 2003; Ottini et al., 2006). Wang et al. identified a novel genomic instability-associated microRNA (miRNA) model that is associated with ovarian cancer prognosis (Wang et al., 2017). A previous study reports that mouse double minute 2 (MDM2) regulates genomic instability and tumorigenesis *via* ubiquitination in human uterine cervix cancer, thyroid cancer, and breast cancer (Cao Z, et al., 2019).

Long non-coding RNAs (lncRNAs) are non-coding RNAs more than 200 nucleotides in length (Jiang et al., 2016); they play important roles in many cellular processes including the survival, proliferation, migration, epigenetic modulation, and chromosomal modification of cells (Meller et al., 2015; Renganathan and Felley-Bosco, 2017; Pang et al., 2019). lncRNAs are closely related to HNSCC initiation and progression (Denaro et al., 2014; Nohata et al., 2016). Recent studies have reported that lncRNAs are involved in regulating genomic instability. For instance, Bao et al. identified two genes and lncRNAs signature that is associated with genomic instability and survival outcomes of breast cancer (Bao et al., 2020). Munschauer et al. found that the lncRNA non-coding RNA activated by DNA damage (NORAD) functions as a topoisomerase complex to prevent genomic instability by regulating the activity of a complex composed of RBMX-TOP1 and other proteins (Munschauer et al., 2018). Moreover, NORAD can maintain genomic integrity through separating PUMILIO proteins from their target mRNAs (Lee et al., 2016). In addition, the lncRNA GUARDIN is necessary for maintaining genomic stability and can prevent chromosome end-to-end fusion through the GUARDIN-miR-23/TRF2 pathway (Hu et al., 2018). The list of lncRNAs involved in the human cancer prognosis is rapidly expanding, but whether genomic instability-associated lncRNAs have a role in predicting the survival outcomes of HNSCC patients has not been fully explored.

With the development of next-generation sequencing technology and high-dimensional datasets, large-scale gene expression studies are now possible. This will enable us to detect aberrantly expressed genomic instability-associated lncRNAs related to cancer occurrence or metastasis and predict patients’ survival probability.

In the present study, we performed a comprehensive analysis of The Cancer Genome Atlas (TCGA) database and explored the effect of genomic instability-associated lncRNAs on the survival of HNSCC patients. A least absolute shrinkage and selection operator (LASSO) regression algorithm was used to analyze high-dimensional data, a four genomic instability-associated lncRNA prognostic model was constructed to generate a prognostic risk score that was used to stratify patients. regression analysis was performed to assess the relationship between the signature’s predictive value and clinical information of HNSCC patients. A nomogram was built to predict the overall survival (OS) of patients with HNSCC. Finally, we validated the expression levels of four genomic instability-associated lncRNAs in HNSCC tissues and adjacent normal tissues with *in situ* hybridization experiments. In summary, we developed a reliable four-lncRNA genomic instability-associated lncRNA signature (GILncSig) related to genomic instability that could function as an independent prognostic marker for HNSCC.

## Materials and Methods

### Data Collection

The gene expression profiles and somatic mutation information of 546 HNSCC patients, and the corresponding clinical data of 528 HNSCC patients were downloaded from The University of California Santa Cruz (UCSC) Xena browser (https://xenabrowser.net/) with cohort name: TCGA-HNSC. Then, the gene expression profiles of the TCGA-HNSC cohort (fragments per kilobase of transcript per million mapped reads values) were transformed into transcripts per kilobase million (TPM) values. Ensemble IDs were converted to gene symbols using the “org.Hs.eg.db” and “clusterProfiler” R packages. Strawberry Perl (version 5.32.0; http://strawberryperl.com/) was used to extract the gene expression data from the TCGA-HNSC cohort and construct a data matrix for further analysis. HNSCC patients with a survival time <30 days or without clinical data were removed to avoid the interference of confounding factors. We employed 491 HNSCC samples with complete survival information, paired lncRNA and mRNA expression profiles, somatic mutation data, and clinical information to analyze genomic instability-associated lncRNAs signatures and construct a prognostic risk model. We randomly divided the 491 HNSCC samples into a training set (246 samples) and a testing set (245 samples) using the “caret” R packages. The training set of 246 samples was used to verify the genomic instability-associated lncRNA signatures and build a prognostic model. The testing set of 245 samples was used to independently validate prognostic risk model performance.

### Identification of lncRNAs Related to Genomic Instability

To identify mutation-derived binding genomic instability-associated lncRNAs, we extracted lncRNA somatic mutation profiles and expression profiles of each sample in the HNSCC cohorts. After calculating the cumulative number of somatic mutations for each individual, patients were sorted in ascending order based on the number of somatic mutations using Wilcoxon rank-sum tests. The top 25% of patients with the highest mutation frequencies were defined as the genomic unstable (GU)-like groups, and the lowest 25% of patients with lowest mutation frequencies were defined as the genomic stable (GS)-like groups. The lncRNA expression profiles of both groups were compared using Wilcoxon rank-sum tests in the “limma” package of R software. Consequently, the differentially expressed lncRNAs [|Fold Change| > 1.0 and false discovery rate (FDR)-adjusted *p* < 0.05] were considered as genomic instability-associated lncRNAs. A volcano plot was constructed to represent differentially expressed lncRNAs between the GU-like and GS-like groups using the “ggpubr” and “ggthemes” packages in R software.

### Hierarchical Clustering Analysis

First, we normalized the expression data of genomic instability-associated lncRNAs from all 491 HNSCC samples using a Z-score analysis. Then we performed hierarchical clustering analyses to classify all samples into two clusters by using the “limma, sparcl and pheatmap” packages in R. According to the somatic mutation counts, the clusters with higher somatic mutation counts were defined as GU-like clusters, whereas the clusters with lower somatic mutation counts were defined as GS-like clusters (Mann–Whitney *U* test, *p* < 0.05).

### Development of the GILncSig

Univariate Cox regression analysis was performed to evaluate correlations between the expression levels of genomic instability-associated lncRNAs and HNSCC patient OS (*p* < 0.001) on the training cohort. Only lncRNAs that showed significantly associations with OS were considered for the subsequent analysis. Then, LASSO regression was used to screen the narrow candidate genes and avoid overfitting by using the “glmnet” and “survival” R packages (Tibshirani, 1997). The penalty parameter lambda was detected by using 10-fold cross-validation (Friedman et al., 2010). The minimum lambda was defined as the optimal value, and we obtained a list of prognostic signatures with correlation coefficients. Next, multivariate Cox regression analysis was used to identify independent prognostic candidates, and a prognostic model of genomic instability-associated lncRNAs was constructed. The GILncSig was developed based on the expression and coefficient of each genomic instability-associated lncRNAs in the model, and the risk score of each patient was calculated with the following equation:

$$\mathrm{GILncSig}\;\mathrm{Risk}\;\mathrm{score}\left(\mathrm{patients}\right)=\sum_{\mathrm{i=}1}^{n}{\mathrm{Expression}}_{\mathrm{GILncSigi}}\times \;{\mathrm{Coefficient}}_{\mathrm{GILncSigi}}\;$$

Here, “*n*” represents the number of prognostic genomic instability-associated lncRNAs and “*i*” is the serial number of each GlncR. Patients with HNSCC were divided into high- and low-risk groups based on the median GILncSig risk score as a cutoff value according to the risk score equation. Kaplan-Meier survival curve analysis was used to compare the prognostic gene signature and OS of the two groups through the “survival” and “survminer” packages in R software (Tian and Zhang, 2018). Time-dependent receiver operating characteristic (ROC) curves were constructed by the “timeROC” package with R, and the areas under the curve (AUCs) were calculated to measure the sensitivity and specificity of the GILncSig prognostic model. The model’s performance was then evaluated in the testing set and the entire TCGA-HNSC set. The univariate and multivariate Cox regression and stratified analyses were used to determine the relationship between GILncSig expression and other key clinical factors. Hazard ratios and 95% confidence intervals were calculated by Cox regression analysis.

### Functional Enrichment Analysis

Pearson correlation analysis was used to measure the correlations between lncRNAs and paired expression of mRNAs, and the top 10 mRNAs were considered as target genes. A lncRNA-mRNA co-expression network was visualized using the “igraph” package in R. To identify the possible functions of genomic instability-associated lncRNAs, Gene Ontology (GO) and Kyoto Encyclopedia of Genes and Genomes (KEGG) enrichment analysis was performed on co-expressed lncRNA-associated mRNA partners. The KEGG results were analyzed and visualized using the “ggplot2” and “clusterProfiler” packages with R. The GO biological process (BP), cellular component (CC), and molecular function (MF) results were visualized using the “cnetplots” package in R. We used *p* < 0.05 and FDR-adjusted *p* < 0.05 as the GO and KEGG enrichment analysis thresholds.

### Clinical Stratification Analysis of the GILncSig Prognostic Value

To test the prognostic value of GILncSig factor in patients with various clinicopathological features, we implemented survival analysis in the whole TCGA set by using the “survival” package in R. Patients with HNSCC were stratified into various subgroups according to clinical parameters, including age (≤65 and >65), sex (female and male), tumor-node-metastases (TMN) classification, and tumor stage (I-II and III-IV). Then patients in each subgroup were divided into high- and low-risk groups based on the median GILncSig risk score.

### Human HNSCC Samples and *in situ* Hybridization Experiments

Ethical approval for this study was obtained from the Medical Ethics Committee of the Second Xiangya Hospital of Central South University (KQ2019FY01). Eleven pairs paraffin specimens of HNSCC and para-carcinoma tissue samples were collected from the Department of Stomatology, the Second Xiangya Hospital, Central South University (Supplementary Table S1). The pathologic diagnosis of HNSCC was confirmed.

The expression levels of the four genomic instability-associated lncRNAs in tissues were measured with digoxigenin-labeled antisense oligonucleotide probes. Each tissue sample was cut into 5-μm-thick section and mounted on glass slides, which were dried overnight at 37°C, dewaxed in xylene, and rehydrated with graded ethanol. The slides were incubated in citrate solution, heated for antigen retrieval, and soaked with proteinase K (20 μg/mL, Servicebio, Wuhan, China). We then added prehybridization solution to each section and incubated them for 1 h at 37°C. Subsequently, the slides were washed with prehybridization buffer for 30 min at room temperature then incubated overnight at 4°C with the following digoxigenin-labeled antisense oligonucleotide probes:

AC023310.4: 5′-GGT​GGC​AAG​ACG​GAA​TAA​GGG​AAA​GGA​GGG-3′;

AC091729.1: 5′-GCC​ACC​CAA​GAG​CGG​GAA​GAC​GGG​GAT​TGT-3′;

LINC01564: 5′-TGC​TAA​ACT​GTC​CAA​GAT​TAT​GAT​GTG​CTG​GGT​GT-3′;

MIR3142HG: 5′-GGC​TAA​GGG​TCT​GAT​AAG​CAA​AGG​GCG​GAA-3′.

The slides were washed three times with 2× saline sodium citrate for 5 min and then incubated with blocking solution (rabbit serum) at room temperature for 30 min. Mouse anti-digoxigenin-labeled horseradish peroxidase (anti-DIG-HRP, Servicebio, Wuhan, China) was added for incubation at 37°C for 40 min, then sections were washed in phosphate-buffered saline (PBS) for 5 min. They were then washed three times with PBS for 5 min each and dyed with a diaminobenzidine (DAB) chromogenic substrate (Dako, Glostrup, Denmark). The nuclei were counterstained with hematoxylin staining solution for 3 min and washed in tap water. Finally, images were obtained by light microscopy (Carl Zeiss, Oberkochen, Germany) and quantified by ImageJ.

### Statistical Analysis

All analyses were performed with R software (version 4.0.4, 64-bit; https://www.r-project.org/) and its appropriate packages. In addition to those noted above, “ggplot2,” “ggpubr,” “limma,” “tidyverse,” “dplyr,” and “plyr” R packages were also used for data analysis and graph plotting. Perl programming language (version 5.34.0, https://www.perl.org/) was used to process data. Kaplan-Meier survival curve analyses and log-rank tests were used to evaluate differences in OS between the high- and low-risk groups. Differences were considered significant at *p* < 0.05.

## Results

### Identification of Genomic Instability-Associated lncRNAs in HNSCC Patients

**FIGURE 1:**
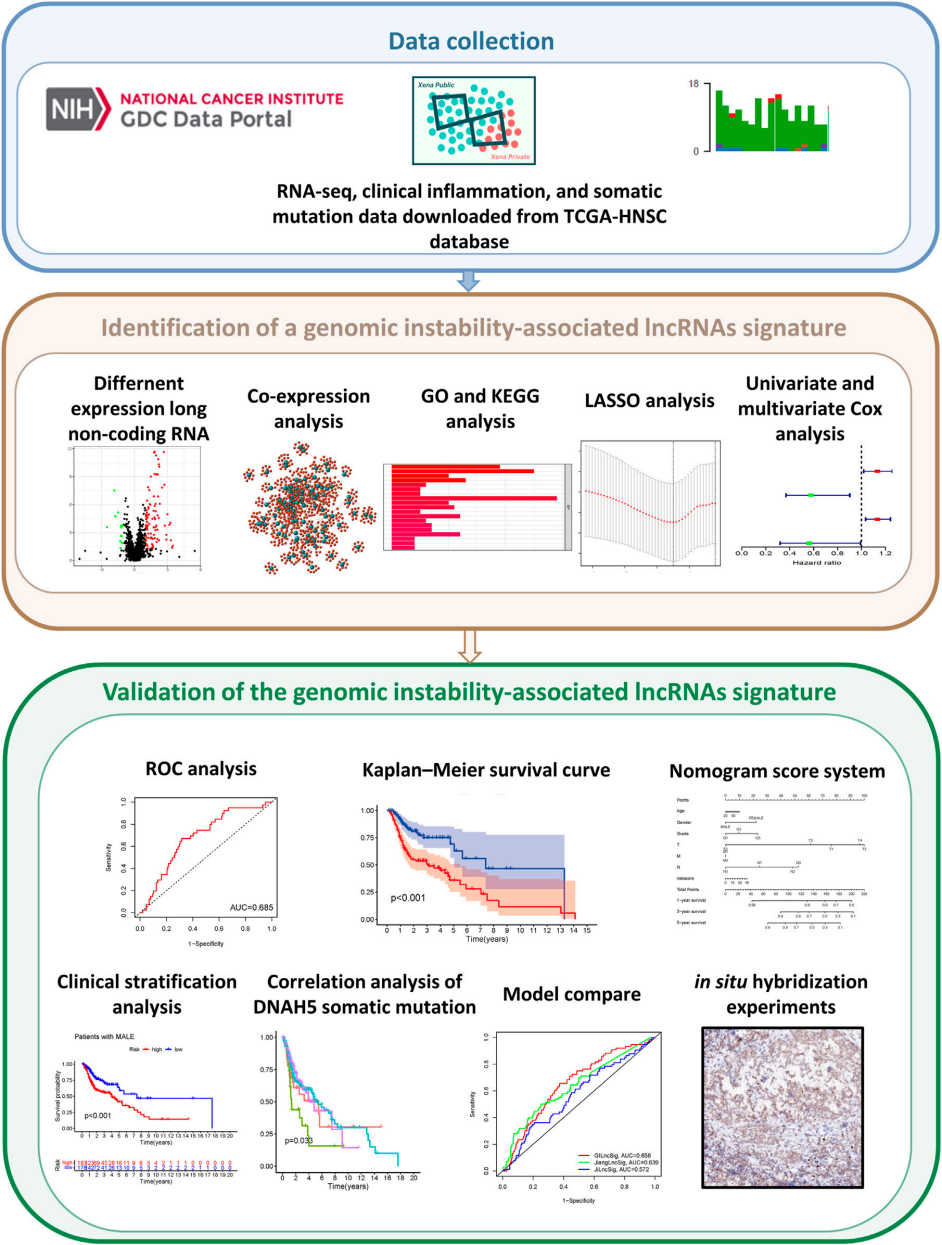
Graphical abstract of genomic instability-associated lncRNAs signature establishment in head and neck squamous cell carcinoma.

**FIGURE 2:**
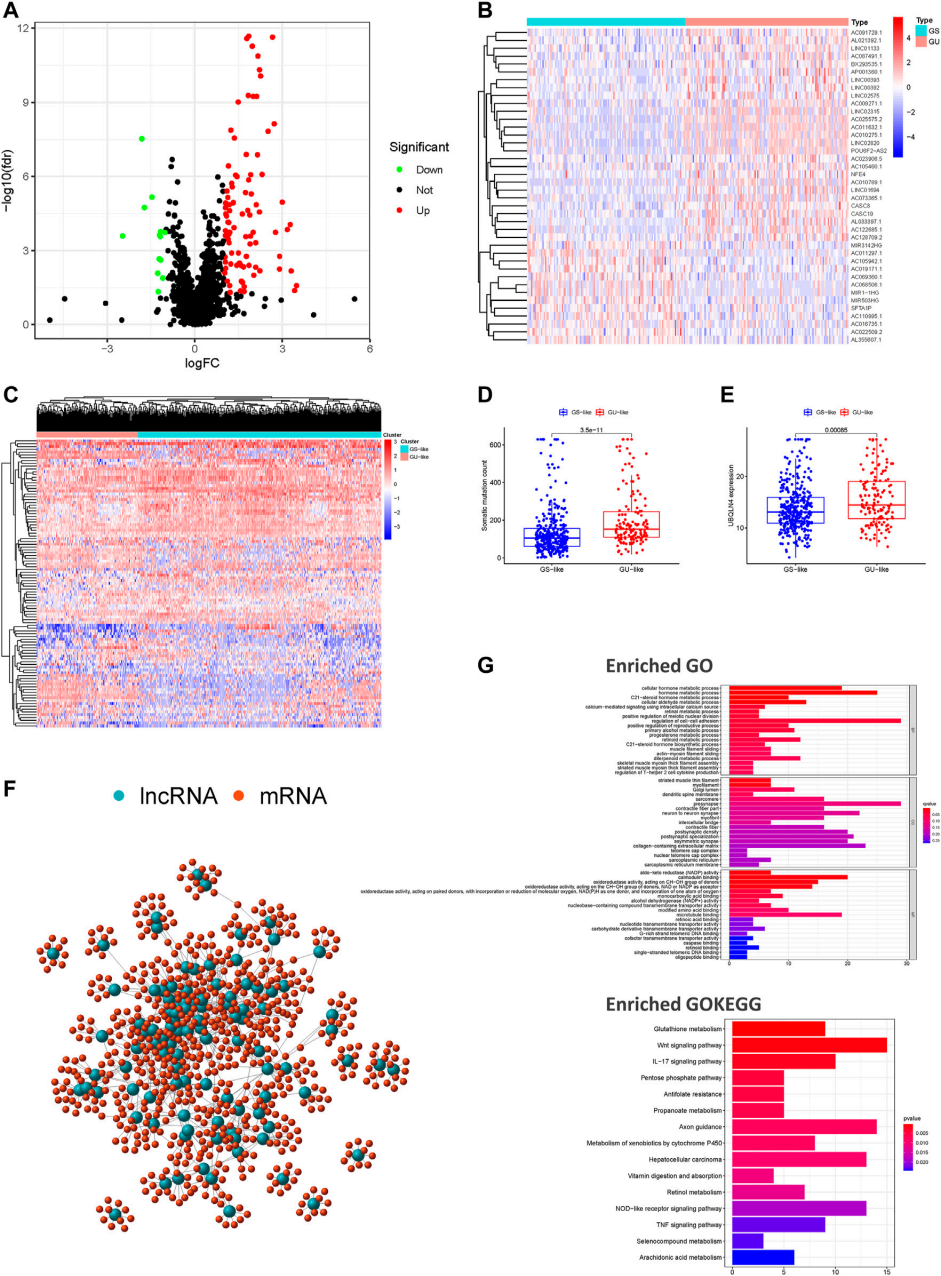
Screening and identifying of genomic instability-related lncRNAs and their functional annotation in patients with HNSCC. **(A)** Volcano plot of 103 differential expressed lncRNAs between the GU-like and GS-like groups. Upregulated lncRNAs are shown in red on the right, whereas downregulated lncRNAs are shown in green on the left. **(B)** Heatmap of expression of the top 50 differential lncRNAs, divided into the GS-like groups (blue) and GU-like group (red). **(C)** An unsupervised clustering of 492 patients with HNSCC was performed based on the expression patterns of 103 candidate genomic instability-associated lncRNAs. The GS-like groups is shown in blue on the right, the GU-like groups is shown in red on the left. **(D)** Boxplots of somatic mutation levels in the GU-like and GS-like groups. **(E)** Boxplots of UBQLN4 expression level in the GU-like and GS-like groups. **(F)** Co-expression networks of differential lncRNAs (blue) and their related mRNAs (red) based on the Pearson correlation coefficients. **(G)** GO enrichment analysis and KEGG pathway analysis of the lncRNA-mRNA network. GO, Gene Ontology; HNSCC, head and neck squamous cell carcinoma; KEGG, Kyoto Encyclopedia of Genes and Genomes.

The study design is depicted in Figure 1. To investigate lncRNAs associated with genomic instability, we sorted them according to the frequency of somatic mutations. We placed the top 25% of somatic mutations (127 samples) into the GU-like groups and the lowest 25% of somatic mutations (123 samples) into the GS-like groups. Then we compared the lncRNA expression profiles of patients in the GU-like and GS-like groups to identify differentially expressed lncRNAs. By using the significance analysis of microarrays method, we screened a total of 103 lncRNAs that were significantly differentially expressed between the two groups, of which 13 lncRNAs were downregulated, and 90 lncRNAs were upregulated in the GU-like groups (|Fold Change| > 1.0, *p* < 0.05, Figure 2A). The clinical and pathological data of HNSCC patients in the GU-like and GS-like groups are listed in Table 1, and differentially expressed lncRNAs are shown in Figure 2B. Then, we applied an unsupervised clustering analysis of the entire TCGA-HNSC cohort based on the levels of 103 differentially expressed lncRNAs. As shown in Figure 2C, all samples were divided into two groups based on the levels of the 103 differentially expressed lncRNAs, and the numbers of mutations in the two groups were significantly different. Notably, the GU-like clusters had a higher number of mutations compared with the GS-like clusters (*p* < 0.001, Mann–Whitney *U* test; Figure 2D). We also compared the expression level of UBQLN4 (a driver gene of genomic instability) between the GU-like clusters and GS-like clusters and found that it was upregulated in the GS-like cluster compared to the GS-like clusters (*p* < 0.001, Mann–Whitney *U* test; Figure 2E).

**TABLE 1:** Clinicopathological information of the three HNSCC patient sets in this study.

Covariates	Type	Total	Testing set	Training set	*p*-value
Age	≤65	321 (65.38%)	164 (66.94%)	157 (63.82%)	0.5279
	>65	170 (34.62%)	81 (33.06%)	89 (36.18%)	–
Gender	FEMALE	130 (26.48%)	63 (25.71%)	67 (27.24%)	0.7796
	MALE	361 (73.52%)	182 (74.29%)	179 (72.76%)	–
Grade	G1	60 (12.22%)	32 (13.06%)	28 (11.38%)	0.2802
	G2	293 (59.67%)	140 (57.14%)	153 (62.2%)	–
	G3	117 (23.83%)	59 (24.08%)	58 (23.58%)	–
	G4	2 (0.41%)	0 (0%)	2 (0.81%)	–
	GX	16 (3.26%)	11 (4.49%)	5 (2.03%)	–
	unknown	3 (0.61%)	3 (1.22%)	0 (0%)	–
Stage	Stage I–II	94 (19.14%)	43 (17.55%)	51 (20.73%)	0.3699
	Stage III–IV	329 (67.01%)	170 (69.39%)	159 (64.63%)	–
	unknown	68 (13.85%)	32 (13.06%)	36 (14.63%)	–
T	T0	1 (0.2%)	0 (0%)	1 (0.41%)	0.7671
	T1–2	173 (35.23%)	85 (34.69%)	88 (35.77%)	–
	T3–4	262 (53.36%)	133 (54.29%)	129 (52.44%)	–
	TX	33 (6.72%)	17 (6.94%)	16 (6.5%)	–
	unknown	22 (4.48%)	10 (4.08%)	12 (4.88%)	–
M	M0	181 (36.86%)	96 (39.18%)	85 (34.55%)	0.5333
	M1	1 (0.2%)	0 (0%)	1 (0.41%)	–
	MX	60 (12.22%)	30 (12.24%)	30 (12.2%)	–
	unknown	249 (50.71%)	119 (48.57%)	130 (52.85%)	–
N	N0	167 (34.01%)	80 (32.65%)	87 (35.37%)	0.5859
	N1–3	300 (61.1%)	153 (62.45%)	147 (59.76%)	–
	unknown	24 (4.89%)	12 (4.9%)	12 (4.88%)	–

### Functional Enrichment Analysis

We performed a lncRNA-mRNA co-expression analysis of the resulting lncRNAs by calculating the Pearson correlation coefficients of each lncRNA-mRNA pair. The correlation coefficients and *p*-values are provided in Supplementary Table S2. As shown in Figure 2F, we constructed a co-expression network of lncRNAs and mRNAs that can reflect the relationships between the two groups. We also conducted GO and KEGG pathway analyses of genomic instability-associated lncRNA-related target genes. We obtained the top 10 most relevant mRNAs among the differential genomic instability-associated lncRNAs in the GS-like and GU-like groups to serve as the target genes. The GO functional analysis of genomic instability-associated lncRNA-related target genes indicated that those mRNAs were mainly involved in the regulation of cell-cell adhesion, striated muscle thin filament, and calmodulin-binding. The KEGG pathway analysis of genomic instability-associated lncRNA-related target genes indicated that those mRNAs were mainly involved in the Wnt signaling pathway, vitamin digestion and absorption, tumor necrosis factor signaling pathway, pentose phosphate pathway, metabolism of xenobiotics by cytochrome P450, metabolic pathway, glutathione metabolism, and biosynthesis of antibiotics (Figure 2G). In total, we identified 103 differentially expressed lncRNAs implicated in genomic instability or destabilization of cellular genomic stability that could disrupt cellular homeostasis and cause an increase in genomic instability. The 103 differentially expressed lncRNAs were defined as genomic instability-associated lncRNAs (Supplementary Table S3).

### GILncSig Development Using the Training Set

**FIGURE 3:**
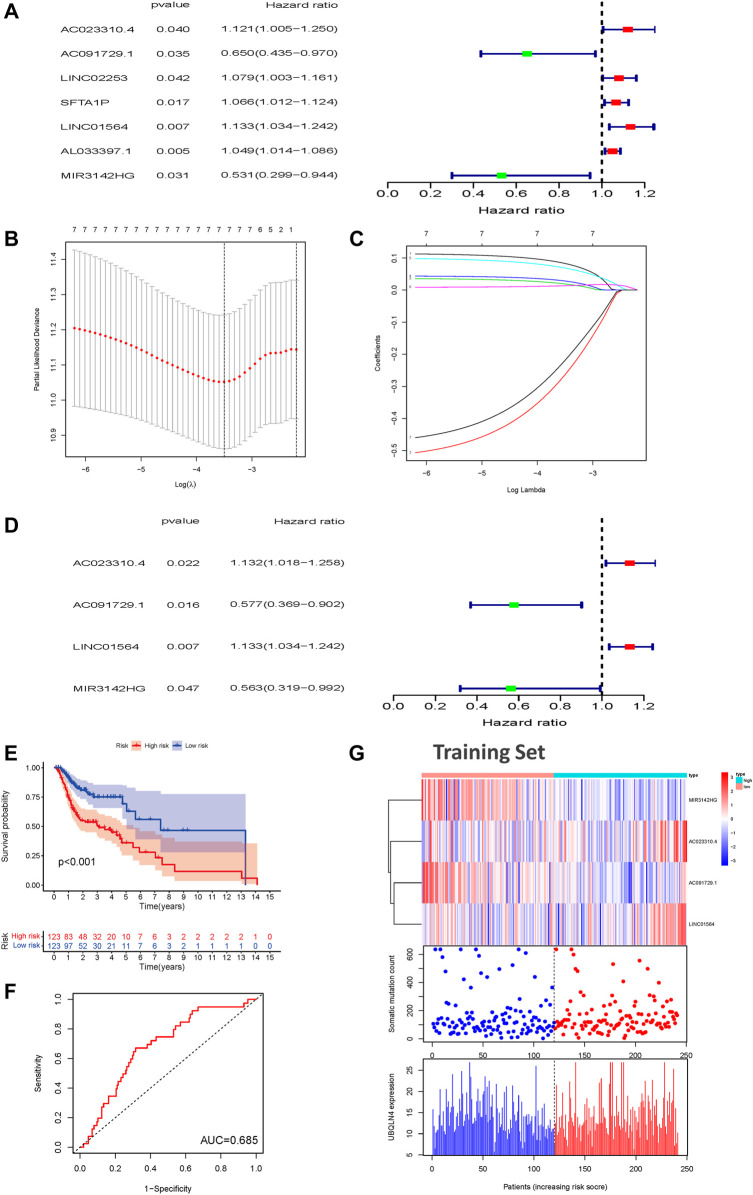
Construction of a prognostic model related to overall survival of HNSCC patients based on genome instability-related lncRNAs in the training set. **(A)** Seven prognostic relevant lncRNAs based on univariate Cox regression analysis. **(B)** Screening the Log Lambda value corresponding to the minimum cross-validation error point. **(C)** The distribution plot of the LASSO coefficient. Selecting genome instability-related lncRNAs with a non-zero coefficient corresponding to the same Log Lambda value. **(D)** Multivariate Cox regression analysis revealed four independent genome instability-related lncRNAs related to patient prognosis. Two lncRNAs were protective (AC023310.4 and LINC01564), and two were risk factors for shorter survival (AC091729.1 and MIR3142HG). **(E)** Kaplan-Meier survival curves for HNSCC patients in the high- and low-risk groups grouped by the GILncSig score in the training set **(F)** Time-independent receiver operating characteristic curves of the GILncSig in the training set. **(G)** LncRNA expression patterns and the distributions of somatic mutations and UBQLN4 expression with increasing GILncSig scores in the training set. GILncSig, genomic instability-associated lncRNAs signature; HNSCC, head and neck squamous cell carcinoma.

To further investigate the predictive prognostic role of the genomic instability-associated lncRNAs, 491 patients from the TCGA-HNSC cohort were randomly divided into a training set (246 patients) and a testing set (245 patients). Univariate Cox proportional hazard regression analysis was performed to identify the relationships of 103 genomic instability-associated lncRNAs with OS in HNSCC patients in the training set. Our results showed that seven genomic instability-associated lncRNAs were significantly associated with the OS of HNSCC patients (*p* < 0.05, Figure 3A). Among the survival-related genes, overexpression of five genomic instability-associated lncRNAs (AC023310.4, LINC02253, SFTA1P, LNC01564, and AL033397.1) was significantly related to worse survival outcomes. In comparison, overexpression of two genomic instability-associated lncRNAs (AC091729.1 and MIR3124HG) showed prognostic value indicating longer OS. We then performed LASSO regression analysis of these genomic instability-associated lncRNAs and calculated regression coefficients (Figure 3B). The LASSO analysis indicated that the model achieved the best performance when it included seven genomic instability-associated lncRNAs (Figure 3C). Finally, multivariate Cox regression analysis was performed to construct the prognostic model, and four genomic instability-associated lncRNAs were identified as independent prognostic factors (Figure 3D). The GILncSig was constructed based on the four genomic instability-associated lncRNAs, and the prognostic risk score was calculated as the following equation: GILncSig Risk score = (expression level of AC023310.4 × 0.12366) + (expression level of AC091729.1 × −0.54962) + (expression level of LINC01564 × 0.125032) + (expression level of MIR3142HG × −0.57480) (Table 2). The coefficients of two lncRNAs (AC023310.4 and LINC01564) were positive in the equation, suggesting that they are risk factors and their overexpression was associated with poor prognosis; the other two lncRNAs (AC091729.1 and MIR3142HG) had negative coefficients and served as protective factors as their upregulated expression was associated with better survival outcomes.

**TABLE 2:** Overall information of four genomic instability-associated lncRNAs significantly associated with the prognosis of patients with head and neck squamous cell carcinoma.

ID	Genomic location	Coefficient	HR	HR.95L	HR.95H
AC023310.4	Chromosome 15q11.2	0.12366	1.13164	1.01812	1.25781
AC091729.1	chromosome 7	−0.54962	0.57717	0.36924	0.90218
LINC01564	chromosome 6p12.1	0.125032	1.13318	1.03404	1.24184
MIR3142HG	chromosome 5q33.3	−0.57480	0.56282	0.31928	0.99211

The risk score of each patient in the training set was calculated according to the prognostic signature. These patients were then divided into high- and low-risk groups based on the median risk score. To assess the survival difference between these two groups, we conducted Kaplan-Meier survival curve analysis. Patients in the high-risk groups show markedly poorer OS than those in the low-risk groups (*p* < 0.001, Figure 3E). Subsequently, the accuracy of the OS estimate derived from the prognostic model was assessed with a time-dependent ROC curve. The AUC of the ROC curve was 0.685 in the training cohort (Figure 3F). A heat map, somatic mutation scatter plot, and gene expression plot were generated to show the relationship between the risk score and somatic mutation pattern of each HNSCC sample in the training set (Figure 3G). Expression of AC023310.4 and LINC01564 in the training set were upregulated in the high-risk groups compared with the low-risk groups, whereas AC091729 and MIR3142HG expression were downregulated in the high-risk groups compared with the low-risk groups.

### GILncSig Validation Using the Testing and TCGA Sets

**FIGURE 4:**
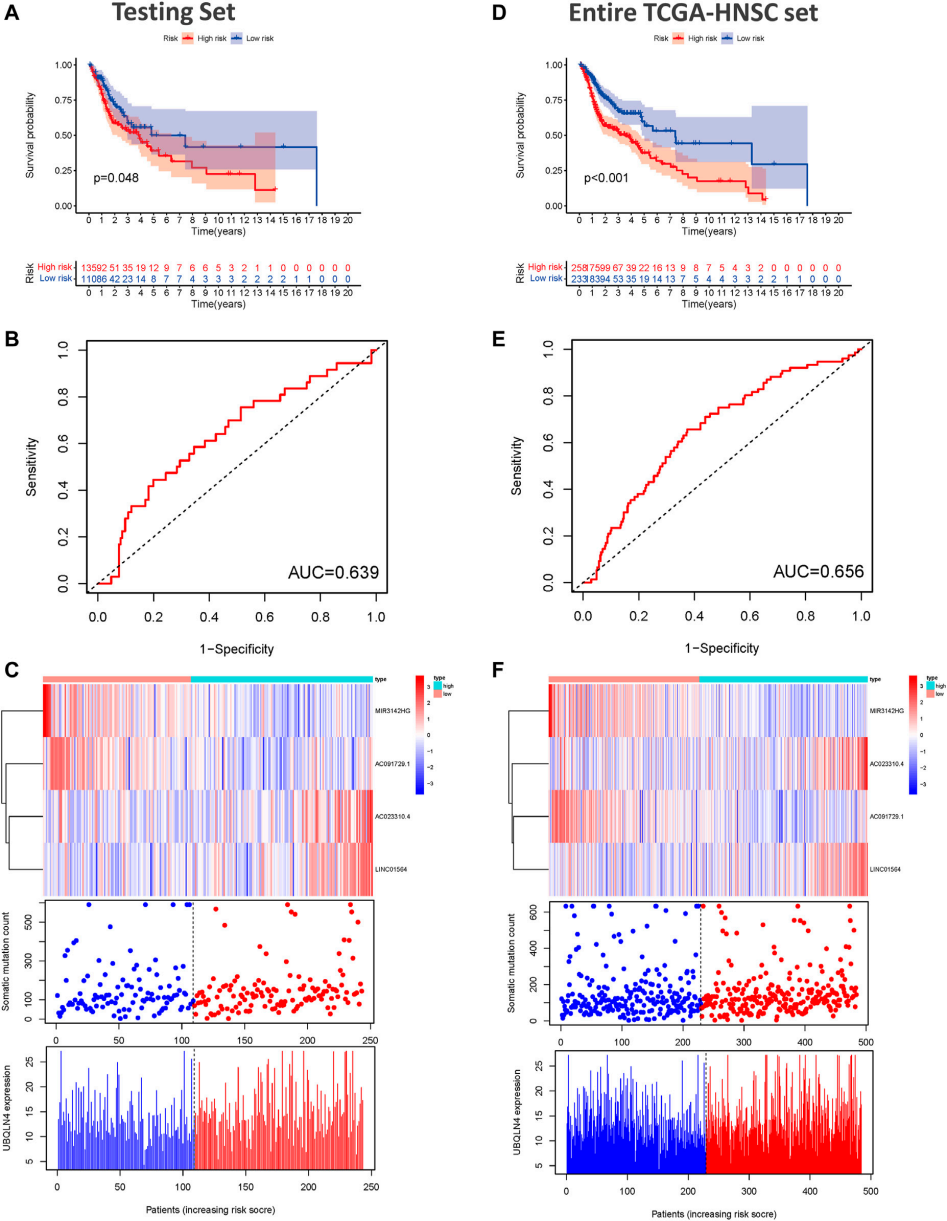
Validation of the predictive performance of the genome instability-related lncRNAs signature in the testing and TCGA sets. **(A)** Kaplan-Meier survival curves for HNSCC patients in the high- and low-risk groups grouped by the GILncSig score in the testing set. **(B)** Time-independent receiver operating characteristic curves of the GILncSig in the testing set. **(C)** lncRNA expression patterns and the distributions of somatic mutation and UBQLN4 expression with increasing GILncSig score in the testing set. **(D)** Kaplan-Meier survival curves for HNSCC patients in the high- and low-risk groups grouped by the GILncSig score in the entire TCGA-HNSC set. **(E)** Time-independent receiver operating characteristic curves of the GILncSig in the entire TCGA-HNSC set. **(F)** LncRNA expression patterns and the distributions of somatic mutation and UBQLN4 expression with increasing GILncSig score in the entire TCGA-HNSC set. GILncSig, genomic instability-associated lncRNAs signature; HNSCC, head and neck squamous cell carcinoma.

To examine the prognostic significance of GILncSig, we investigated its utility in the testing set and entire TCGA-HNSC set. According to the same GILncSig and risk score thresholds derived from the training set, patients in the testing set and entire TCGA-HNSC set were classified into low- and high-risk groups. Kaplan-Meier survival curve analysis showed that patients in the high-risk groups showed markedly poorer OS than those in the low-risk groups (*p* < 0.001, Figure 4A). An unfavorable OS outcome was also seen in the high-risk groups of the entire TCGA-HNSC set (*p* < 0.001, Figure 4B). The areas under the time-dependent ROC curve of the testing set and entire TCGA-HNSC set were 0.639 (Figure 4C) and 0.656 (Figure 4D), respectively. The heat map, somatic mutation scatter plot, and gene expression plot of the GILncSig signature are shown in Figures 4E,F.

### Clinical Stratification Analysis and Independent Prognostic Analysis of the GILncSig

**FIGURE 5:**
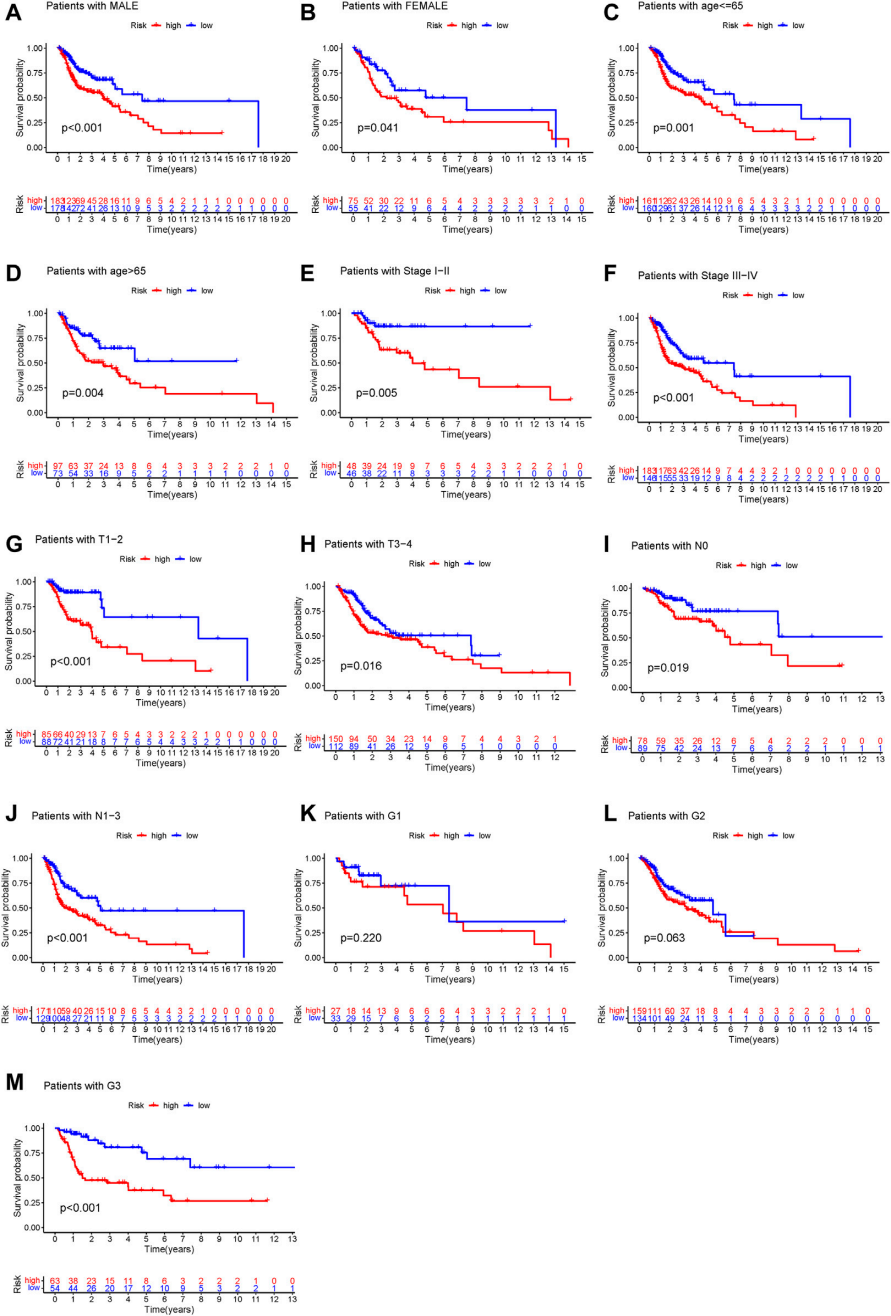
Stratification analysis of the genome instability-related lncRNAs signature. **(A–M)** Kaplan-Meier analysis of clinical subgroups based on the GILncSig scores. The clinical characteristics including: male **(A)**, female **(B)**, age ≤65 **(C)**, age >65 **(D)**, stage I–II **(E)**, stage III–IV **(F)**, T1–2 **(G)**, T3–4 **(H)**, N0 **(I)**, N1–3 **(J)**, G1 **(K)**, G2 **(L)**, and G3 **(M)**.

To determine whether the prognostic value of GILncSig was independent of various clinical subgroups, Kaplan-Meier survival curve analysis was performed to determine the relationship between OS rates in different clinical subgroups of patients according to the risk score level and clinical characteristics such as different stages, age, grade, sex, and TMN status. The results indicated that GILncSig significantly distinguished the prognosis of patients with the following characteristics: female, male, age ≤65, age >65, stage I-II, stage III-IV, G1, G2, G3, T1-2, T3-4, N0, and N1-3, respectively (Figure 5A–M). Based on the median GILncSig score, patients in each clinical subgroup were classified into high- or low-risk groups. We found that clinical subgroups of patients in the low-risk groups had better outcomes than those in high-risk groups. Univariate and multivariate Cox regression analyses of age, sex, tumor stage, tumor stage, and GILncSig risk score were performed to evaluate the independent prognostic value of the GILncSig. Our results suggested that the novel prognostic model could be an independent prognostic factor related to the OS rate of HNSC patients (Table 3). Univariate Cox regression analysis indicated that GILncSig risk score, age, sex, and tumor stage were significantly correlated with the OS rate of HNSC patients (*p* < 0.05). Multivariate Cox regression analysis indicated that age, sex, and tumor stage were significantly correlated with the OS rate of HNSC patients (*p* < 0.05). Together, these findings suggest that the GILncSig was an independent prognostic factor in predicting HNSCC patient survival.

**TABLE 3:** Univariate and multivariate Cox regression analysis of the genomic instability-associated lncRNAs signature and overall survival in different HNSCC patient sets.

Variables	Univariable model				Multivariable model			
	HR	HR.95L	HR.95H	*p*-value	HR	HR.95L	HR.95H	*p*-value
Training set (n = 246)								
Age	1.017	0.996	1.039	0.115	–	–	–	–
Gender	0.931	0.569	1.525	0.777	–	–	–	–
Grade	1.148	0.815	1.618	0.429	–	–	–	–
Stage	1.225	0.952	1.577	0.114	–	–	–	–
GILncSig riskScore	1.453	1.221	1.728	0.000	1.453	1.221	1.728	0.000
Testing set (n = 245)								
Age	1.020	1.000	1.041	0.053	–	–	–	–
Gender	0.618	0.388	0.985	0.043	0.536	0.334	0.859	0.010
Grade	1.036	0.732	1.466	0.841	–	–	–	–
Stage	1.560	1.153	2.110	0.004	1.635	1.209	2.211	0.001
GILncSig riskScore	1.012	0.902	1.136	0.837	–	–	–	–
TCGA-HNSC set (n = 491)								
Age	1.019	1.005	1.034	0.009	1.024	1.008	1.039	0.003
Gender	0.757	0.541	1.059	0.104	–	–	–	–
Grade	1.095	0.859	1.396	0.462	–	–	–	–
Stage	1.365	1.128	1.652	0.001	1.412	1.163	1.715	0.000
GILncSig riskScore	1.071	0.990	1.158	0.086	–	–	–	–

### Establishment and Calibration of an Integrated Nomogram

**FIGURE 6:**
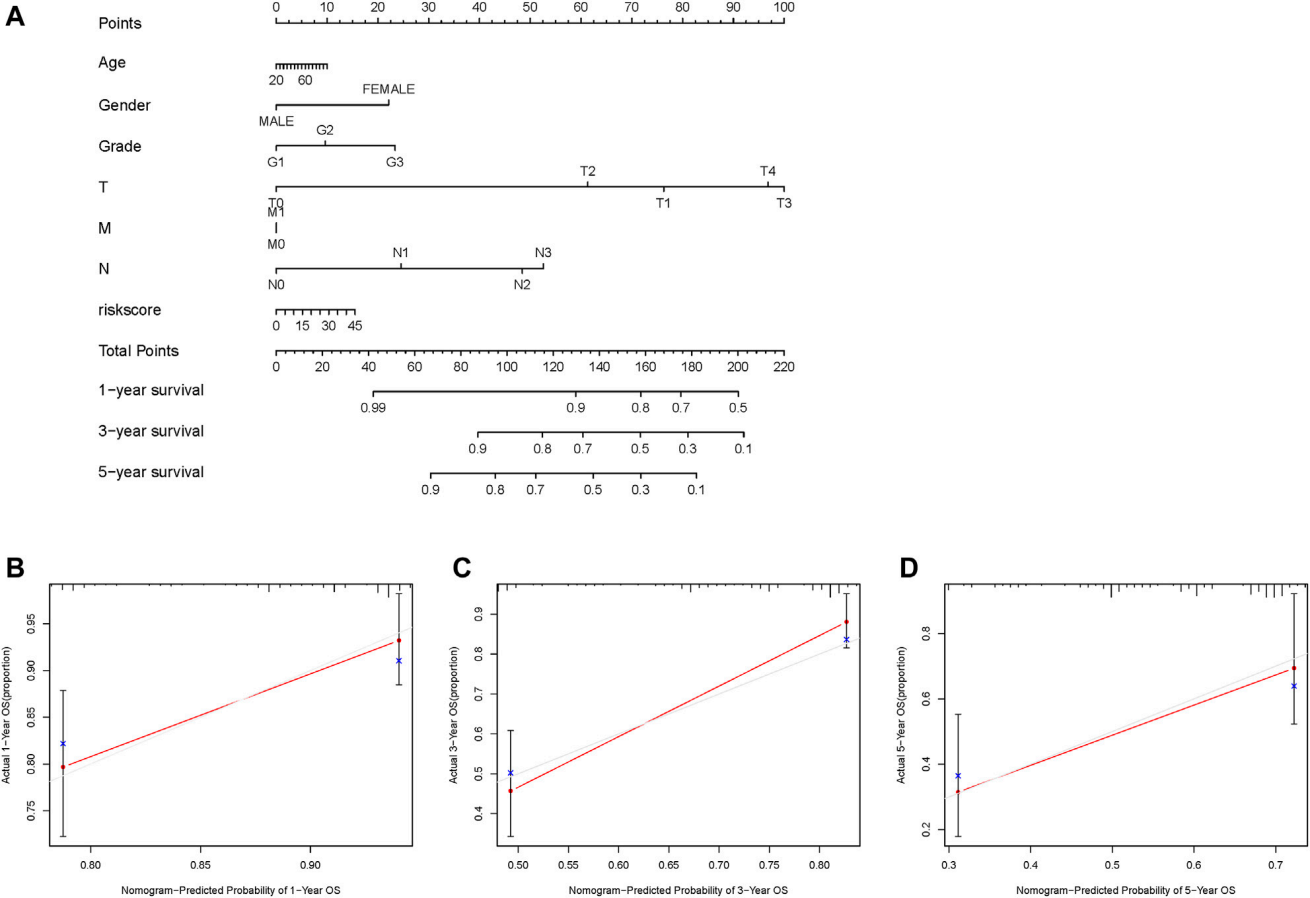
Construction and evaluation of a nomogram based on the genome instability-related lncRNAs signature in the TCGA-HNSC cohort. **(A)** Development of a nomogram based on the GILncSig score. **(B–D)** Calibration plots for the signature at 1, 3, and 5 years. GILncSig, genomic instability-associated lncRNAs signature.

A nomogram was constructed based on age, sex, grade, TMN status, and GILncSig risk score to predict the 1-, 3-, and 5-years survival rates (Figure 6A). A calibration curve was used to evaluate the predictive value of the nomogram. The results indicated optimal agreement between the nomogram-predicted and observed OS rates (Figure 6B), suggesting that the GILncSig had good predictive value for patients with HNSCC.

### Correlation of the GILncSig with DNAH5 Somatic Mutations

**FIGURE 7:**
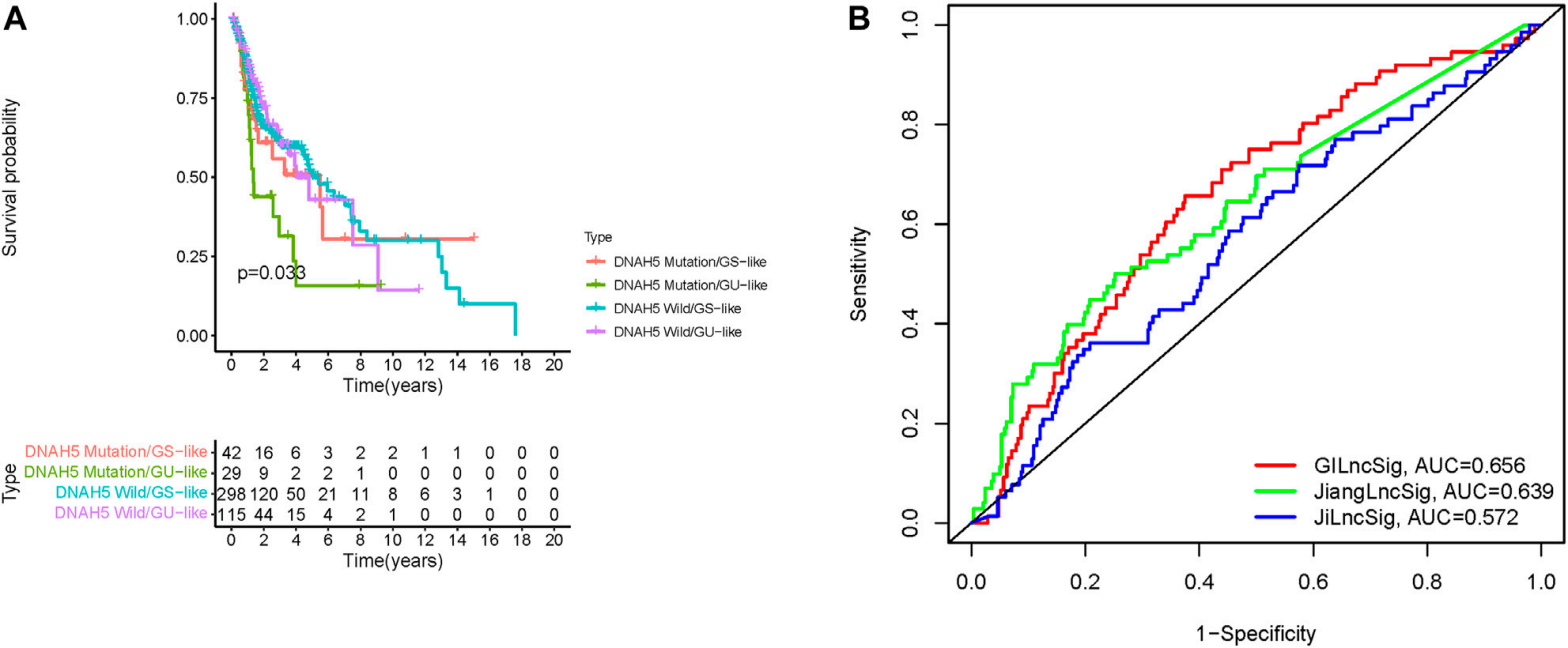
Relationship between the GILncSig and DNAH5 somatic mutation and model comparison. **(A)** Kaplan-Meier curve analysis of overall survival of patients with DNAH5 mutant or wild-type status for the combined GS-like and GU-like groups. **(B)** Time-independent receiver operating characteristic curves of overall survival for GILncSig, Jiang’s LncSig, and Ji’s LncSig. GILncSig, genomic instability-associated lncRNAs signature; DNAH5, dynein axonemal heavy chain 5.

A previous study reported that dynein axonemal heavy chain 5 (DNAH5) mutation was associated with poor survival of patients with esophageal squamous cell carcinoma (Qing et al., 2017). Therefore, we analyzed the prognosis performance of the GILncSig combined with DNAH5 mutation status. We compared survival differences between the GU-like and GS-like groups in the DNAH5 mutation status subgroup using the log-rank tests. HNSCC patients were grouped into four groups: DNAH5 Mutation/GS-like groups, DNAH5 Mutation/GU-like groups, DNAH5 Wild/GS-like groups, and DNAH5 Wild/GU-like groups (*p* < 0.001, Figure 7A). The DNAH5 Wild/GU-like groups had a better OS rate than the DNAH5 Mutation/GU-like groups, and patients in the DNAH5 Mutation/GS-like groups had a higher OS rate. These findings indicated that GILncSig combined with DNAH5 mutation status has good prognostication performance.

### Comparison of the GILncSig with Existing lncRNA-Related Signatures

We next compared the predictive performances of our prognostic model and two lncRNAs signatures previously developed based on the same TCGA-HNSC cohort. Jiang et al. (2021), and Ji and Xue (2020) generated signatures based on three and four novel lncRNAs, respectively. As depicted in Figure 7B, the AUC for the 1-year survival rate of our genomic instability-associated lncRNA prognostic model was 0.656, which was significantly higher than Jiang’s LncSig (AUC = 0.639) and Ji’s LncSig (AUC = 0.572). These results demonstrated the better credibility and effectiveness of our GILncSig in predicting the prognosis of HNSCC patients.

### Expression Analysis of the GILncSig in Tumor Tissues

**FIGURE 8:**
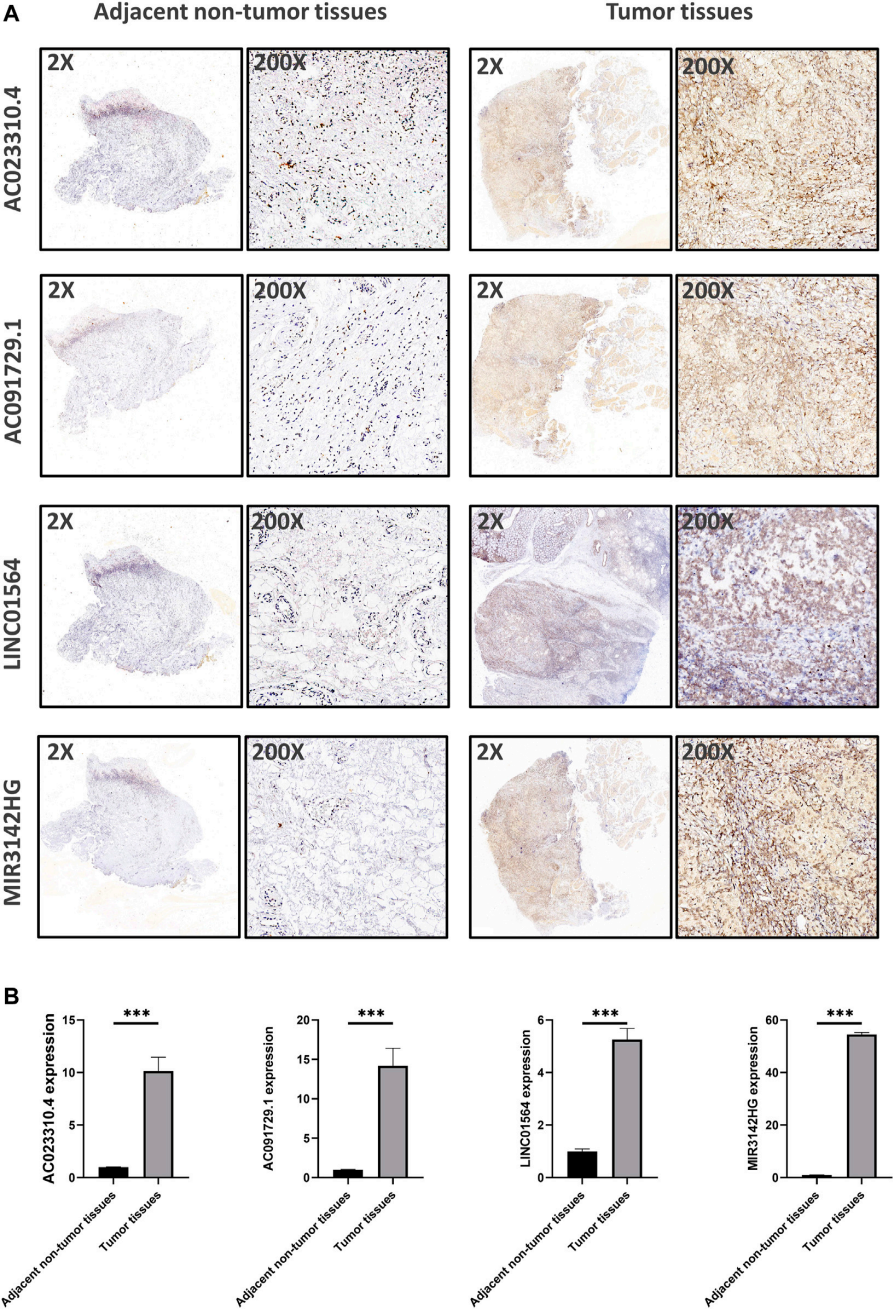
Verification of the expression levels of the genomic instability-associated lncRNAs signature in clinical samples. **(A)** Representative images of *in situ* hybridization experiments in HNSCC patients. Nucleus stained with hematoxylin appear blue, and positive expression of DAB is brownish yellow. **(B)** Relative expression of the four lncRNAs in HNSCC patients.

Using *in situ* hybridization experiments, we next analyzed the expression of the genomic instability-associated lncRNA prognostic signature in tumor samples from patients. HNSCC tissues were matched with adjacent non-tumor tissues were used to verify the differential expression levels of all four lncRNAs in the genomic instability-associated lncRNA prognostic signature. As shown in Figure 8, AC023310.4, AC091729.1, LINC01564, and MIR3142HG were expressed at higher levels in tumor samples compared to adjacent non-tumor tissues.

## Discussion

In recent years, personalized treatment consisting of surgery followed by immune checkpoint inhibition for advanced HNSCC has increased the patient survival rate (Johnson et al., 2020). However, HNSCC is a complex and heterogeneous tumor characterized by multiple genetic mutations, epigenetic alterations, DNA damage repair, and chromosomal deletions. Accumulating evidence shows that survival outcomes vary greatly among HNSCC patients due to limitations of traditional clinicopathological features, especially in advanced-stage disease (Ang et al., 2010; Ramos et al., 2010). It is crucial to identify novel biomarkers to predict clinical outcomes. Genomic instability plays essential and dominant roles in facilitating tumor progression and recurrence, which may have potential diagnostic and prognostic value for cancer patients (Shen, 2011; Duijf et al., 2019). The main sources of genomic instability are DNA damage and aberrant transcriptional or epigenetic changes (Ferguson et al., 2015). However, accurate quantitative measures to describe the degree of genomic instability have not been fully elucidated. Efforts are ongoing to explore the potential relationship between protein-coding genes or miRNAs and genomic instability (Habermann et al., 2009; Mettu et al., 2010; Ferguson et al., 2015).

lncRNAs, a novel class of ncRNAs, are an essential component of tumor biology, and their dysfunction has been related to cancer initiation and progression, including bladder cancer, pancreatic cancer, glioma, and breast cancer (Awasthee et al., 2018; Deng et al., 2018; Fu et al., 2018; Cao H. L. et al., 2019). In addition, lncRNAs may be useful prognostic markers as they are correlated with the prognosis of many different types of tumors (Prensner et al., 2011). Recent advances in understanding lncRNA characteristics revealed a close association between lncRNAs and genomic instability. Munschauer et al. suggested that NORAD plays an important role in maintaining genomic instability (Munschauer et al., 2018); however, the effect of genomic instability-associated lncRNAs on the prognosis of patients with HNSCC remains unknown. A computational frame was recently constructed to analyze correlations between lncRNA expression levels and somatic mutation phenotypes (Bao et al., 2020). The aim of this study was to construct a genomic instability-associated lncRNAs signature to determine its prognostic value in HNSCC patients.

We screened 103 novel lncRNAs that affect HNSCC genomic stability using a mutator hypothesis-derived computational method to develop a model containing four genomic instability-associated lncRNAs. AC023310.4, AC091729.1, LINC01564, and MIR3142HG, were identified in the training set. These genomic instability-associated lncRNAs are closely associated with the OS and clinical outcome of HNSCC patients and take part in many KEGG pathways that correlated with tumor development and progression. According to the GILncSig risk score, HNSCC patients were grouped into low- and high-risk groups with statistically significant differences in survival outcomes. The Kaplan-Meier analysis showed that the OS of patients in the low-risk groups was significantly longer compared with patients in the high-risk groups. The testing set data were used to assess the prognosis risk of patients based on the GILncSig risk score. Kaplan-Meier survival curve analysis showed that the GILncSig also had good performance in patient prognosis classification in the testing set. The nomogram plot showed that the GILncSig was a good predictor for the prognosis outcomes of HNSCC patients. Nomogram calibration revealed good agreement between the predicted and observed OS rates. In addition, the univariate and multivariate Cox regression analyses showed that the GILncSig was an independent and accurate prognostic factor for patients with HNSCC. Notably, the GILncSig was a robust prognostic factor of other clinicopathological characteristics.

Four genomic instability-associated lncRNAs (AC023310.4, AC091729.1, LINC01564, and MIR3142HG) were selected as the prognostic signature in this study. Specifically, AC023310.4 and LINC01564 were risk factors for survival, whereas AC091729.1and MIR3142HG were protective factors for patient prognosis. A careful literature search revealed that AC023310.4 which located on chromosome 15q11.2 was first reported here, and its biological function has not been reported to date. AC091729.1 is located on chromosome 7, Yu et al. identified another version AC091729,7 plays a carcinogenic role and serves as a novel biomarker and latent curative target in sinonasal squamous cell carcinoma patients (Yu et al., 2019). LINC01564 is located on chromosome 6p12.1, Zhang et al. reported that LINC01564 was associated with hepatocellular carcinoma cell survival. It can attenuate the inhibitory effect of miR-107/103a-3p on phosphoglycerate dehydrogenase gene expression through endogenous competitive sponging of miR-107/103a-3p, thus producing a carcinogenic factor in hepatocellular carcinoma (Zhang et al., 2021). In addition, Ke et al. found that high LINC01564 expression was associated with poor OS of patients with testicular cancer (Ke et al., 2021). MIR3142HG, located on chromosome 5q33.3, is correlated with glioma prognosis in the Chinese Han population (Guo et al., 2020).

We also analyzed the correlation between DNAH5 mutation status combined with genomic instability-associated lncRNAs and prognostic outcomes. Based on the Kaplan-Meier survival curve analysis, the prognosis outcome hierarchy was DNAH5 Wild/GS-like groups > DNAH5 Wild/GU-like groups > DNAH5 Mutation/GS-like groups > DNAH5 Mutation/GU-like groups (*p* < 0.05). The results suggest that patients with DNAH5 Mutation in the GS-like groups had better survival outcomes than patients with DNAH5 Mutation in the GU-like groups. DNAH5 mutations are common in patients with esophageal squamous cell carcinoma and are associated with poor survival (Mangalaparthi et al., 2020; Ma et al., 2021). In addition, Li et al. reported that DNAH5 was a novel oncogenic driver in human lung squamous cell carcinoma (Li et al., 2016). The significant difference in survival outcome of TP53 mutation statuses between the GS-like and GU-like groups suggested that DNAH5 mutation combined with genomic instability-associated lncRNAs was an effective prognostic indicator.

This study has several limitations. First of all, the GILncSig was constructed and validated in the TCGA database, therefore more independent datasets are needed to validate our findings. In addition, the platform used for the HNSCC cohort in the Gene Expression Omnibus database does not contain the above four genomic instability-associated lncRNAs. Secondly, the molecular mechanisms of these four lncRNAs function require further *in vitro* or *in vivo* study. Finally, although GILncSig expression levels were validated in tumor tissues from HNSCC patients treated at our hospital through *in situ* hybridization experiments, larger clinical cohorts are needed to validate the predictive accuracy of GILncSig.

In summary, we identified an independent and robust prognostic risk model comprising four genomic instability-associated lncRNAs. This model can effectively predict the OS of HNSCC patients and assess genomic instability. The *in situ* hybridization experiments confirmed differential expression of all four lncRNAs between adjacent non-tumor and tumor tissues from HNSCC patients. Our results show that the four lncRNAs are useful indicators that could affect clinical subgroup management and predict the prognosis of patients with HNSCC.

## Data Availability

The datasets presented in this study can be found in online repositories. The names of the repository/repositories and accession number(s) can be found in the article/Supplementary Material.
