# Supplementary material for: Identification and Validation of a Novel Genomic Instability-Associated Long Non-Coding RNA Prognostic Signature in Head and Neck Squamous Cell Carcinoma
Source: Front Cell Dev Biol. 2022 Jan 20;9:787766. doi: 10.3389/fcell.2021.787766 (PMC8812830; doi:10.3389/fcell.2021.787766)
Supplement: Supplementary file 3 [file DataSheet1.DOCX]

| **Baseline characteristics** | **Value^*^** |
| --- | --- |
| **Age (years)** | 54.6 (44-66) |
| **Gender** |  |
| Male | 8 (73) |
| Female | 3 (27) |
| **Smoking status** |  |
| Yes | 7 (64) |
| No | 4 (36) |
| **Grade** |  |
| G1 | 2 (18) |
| G2 | 5 (45) |
| G3 | 3 (27) |
| G4 | 1 (10) |
| GX | 0 |
| **Stage** |  |
| I- II | 3 (27) |
| III- IV | 6 (55) |
| unknow | 2 (18) |

* values are expressed as median (range) or n (%).
